# Interferon Epsilon Signaling Confers Attenuated Zika Replication in Human Vaginal Epithelial Cells

**DOI:** 10.3390/pathogens11080853

**Published:** 2022-07-29

**Authors:** James W. Mungin, Xin Chen, Bindong Liu

**Affiliations:** Centers for AIDS Health Disparity Research, Department of Microbiology, Immunology, and Physiology, Meharry Medical College, Nashville, TN 37208, USA; jmungin15@email.mmc.edu (J.W.M.J.); xchen@mmc.edu (X.C.)

**Keywords:** Zika virus, sexual transmission, interferon epsilon, human vaginal epithelial cells, primary cervical cell

## Abstract

Zika virus (ZIKV) is an emerging flavivirus that causes congenital birth defects and neurological compilations in the human host. Although ZIKV is primarily transmitted through infected mosquitos, recent studies reveal sexual contact as a potential transmission route. In vagina-bearing individuals, the vaginal epithelium constitutes the first line of defense against viruses. However, it is unclear how ZIKV interacts with the vaginal epithelium to initiate ZIKV transmission. In this study, we demonstrate that exposing ZIKV to human vaginal epithelial cells (hVECs) resulted in de novo viral RNA replication, increased envelope viral protein production, and a steady, extracellular release of infectious viral particles. Interestingly, our data show that, despite an increase in viral load, the hVECs did not exhibit significant cytopathology in culture as other cell types typically do. Furthermore, our data reveal that the innate antiviral state of hVECs plays a crucial role in preventing viral cytopathology. For the first time, our data show that interferon epsilon inhibits ZIKV replication. Collectively, our results in this study provide a novel perspective on the viral susceptibility and replication dynamics during ZIKV infection in the human vaginal epithelium. These findings will be instrumental towards developing therapeutic agents aimed at eliminating the pathology caused by the virus.

## 1. Introduction

Zika virus (ZIKV) is a pathogenic, enveloped RNA virus associated with severe fetal defects and neurological complications in humans [1,2,3,4,5,6,7,8,9,10,11,12,13]. This emerging virus belongs to the flavivirus genus, which consists of globally relevant viruses including Dengue virus, West Nile virus, Hepatitis C, and many others. In the majority of clinical cases, ZIKV-infected patients are asymptomatic for clinical ZIKV disease [14,15,16]. Symptomatic cases exhibit mild febrile illness accompanied by conjunctivitis, headache, arthralgia, skin rash, and muscle and joint pain. Apart from these clinical cases, a subset of fetal and adult patients exhibited neurological complications that included microcephaly and Guillain-Barré Syndrome (GBS), respectively. Microcephaly is a neurological malformation in newborns characterized by abnormal smallness of the head due to incomplete brain development. In addition to microcephaly, other forms of ZIKV-associated congenital disease were present among pregnant patients, including spontaneous abortion, stillbirth, placental deficiency, and cerebral calcification [16]. During the recent ZIKV outbreak, ZIKV was linked to increased cases of GBS, a rare neurological disorder in which the immune system undergoes an autoimmune response and attacks the myelin sheaths of motor neurons. Individuals impacted by GBS experience limb weakness, paralysis, and, in severe chronic cases, impairment of involuntary muscles. The devastating effects of ZIKV clinical disease and its impact on developing fetuses garnered international concern, thus warranting further investigation into this pathogen.

Unlike most mosquito-borne flaviviruses, ZIKV has been discovered to be sexually transmissible and may contribute to clinical disease [17,18,19,20,21,22,23,24,25,26,27,28,29]. The World Health Organization has reported that multiple countries had confirmed evidence of transmission via sexual contact with an infected partner [30]. These sexually transmitted ZIKV cases were described among symptomatic and asymptomatic patients who engaged in coitus via anogenital, oral, and intravaginal routes. Although sexual transmission accounts for a small percentage of total ZIKV cases, recent studies confirm that sexual contact is a relevant transmission route for two major reasons. Firstly, clinical reports show that ZIKV-infected patients exhibited persistent viral shedding in both vaginal fluids and semen [18,19,20,23]. This may play a critical role in spreading the virus into vulnerable areas regardless of mosquito vector distribution and/or environmental conditions. Secondly, numerous in vivo and in vitro studies have validated that the tissues that make up the genital tract support active ZIKV replication [31,32,33,34,35,36,37,38,39,40,41,42,43,44,45,46,47,48,49,50,51,52,53,54,55,56]. Most importantly, animal studies showed that intravaginal ZIKV infection resulted in vaginal transmission, fetal brain infection, enhanced in utero transmission, placental dysfunction, and fetal growth restriction [34,51,56,57,58,59]. Despite growing evidence implicating ZIKV vaginal transmission, the molecular basis of ZIKV pathogenesis in the human vaginal epithelium remains elusive.

The human vaginal tract (HVT) serves as both a mucosal barrier and an entry point for several sexually transmitted viral infections [60,61]. HVT consists of distinct, multiple epithelial cell layers and a mucus membrane lining the epithelial layer. This structure provides a mechanical and physical barrier against foreign pathogens. Despite its protective properties, many sexually transmitted viruses exploit HVT as a portal of entry through several invasion mechanisms. For example, one study showed that human immunodeficiency virus 1 (HIV-1) utilizes the recycling pathway to transport across the vaginal epithelial layer and facilitate vaginal transmission [62]. Furthermore, herpes simplex virus types 1 and 2 (HSV-1 and HSV-2) directly infect the epithelial layer of a variety of mucosal surfaces, including the HVT [63,64]. Interestingly, human papilloma virus (HPV) uses a unique invasion mechanism that involves infecting the mucosal epithelial layer through the basement membrane, which requires disruption of epithelial cell integrity [65,66,67]. In the context of ZIKV, the molecular interactions between the virus and the human vaginal epithelia warrant further investigation.

Type I IFNs play a critical role in the induction of antiviral immune responses during infection, including flaviviruses [31,63,68,69,70,71,72,73]. During infection, permissive cells express soluble glycoproteins from the type I IFN family (IFN α, β, κ, ε, ω) that restrict viral replication through inducible and non-inducible means. ZIKV, however, has been shown to impair those innate antiviral activities by antagonizing type I IFN production and signaling [71,73,74]. Recent studies confirmed that ZIKV infection suppresses type I IFN induction by downregulating IRF3- and NK-κB-mediated signaling [71]. Additionally, ZIKV nonstructural protein 5 (NS5) has been shown to interfere with IFN-mediated signaling by degrading the transcription factor STAT2 [72,74]. Collectively, these studies underscore the mechanism by which ZIKV evades innate immune response among various human cell types, which can result in lytic infection. Despite the antagonistic mechanisms that target conventional type I IFNs (IFN α and β), various cell types express other type I IFNs that confer innate protection against viral infections, including interferon epsilon (IFNε) [75,76,77,78,79,80,81,82,83,84,85,86,87,88]. IFNε is a unique type I IFN that is constitutively expressed in the small intestine, brain, lung, and reproductive tissues. Interestingly, IFNε has been shown to be substantially expressed in the ovarian tissues, uterus, cervix, and vagina [77,81,86,88]. The signaling and bioactivity (immunoregulation, antiviral activity, and antiproliferation) are described to be hormonally regulated and not induced by known PRR pathways, unlike the conventional IFNs α and β. A study by Bourke et al. characterized the spatiotemporal expression of IFNε under conditions of sex hormone stimulation. Their results concluded that IFNε expression significantly decreased following progesterone exposure in contrast to estrogen, where IFNε expression did not undergo any significant change. It is important to note, however, that sex hormones regulated IFNε expression exclusively in the endometrium, despite high constitutive expression in the vagina and other tissue types. Apart from its reproductive role, IFNε plays a pivotal part in mucosal immunity among various mucosal cell types. Studies show that IFNε provides a sustainable antiviral state that controls the proliferation of foreign pathogens invading the genital tract [75,76]. Interestingly, one study highlights the utility of IFNε as a vaccine adjuvant that transiently induces a localized mucosal immune response [75,84]. Another study from Fung et al. demonstrated that IFNε-deficient mice exhibited increased susceptibility to common sexually transmitted infections in the vaginal tract, including herpes simplex virus 2(HSV-2) and *Chlamydia muridarum* [89]. Unfortunately, there is limited evidence to further address the antiviral role of IFNε against other emerging sexually transmitted viral infections, such as ZIKV. Considering ZIKV as a sexually transmitted flavivirus, it is important to examine the antiviral role of IFNε during ZIKV infection in human vaginal epithelial cells.

Considering the nature of vaginal intercourse, the potential target cells for ZIKV vaginal transmission are likely to be localized to the outermost epithelial layer in the HVT. Given that, we hypothesized that the initial event for ZIKV infection during vaginal transmission most likely occurs through direct viral replication of the epithelial cell layer. In this study, we demonstrated that human vaginal epithelial cells (hVECs) are a direct cell target in initiating active ZIKV replication and production during vaginal transmission. Most importantly, the innate antiviral state of hVECs via IFN signaling contributes to both viral attenuation and conferment of viral spread during the event of vaginal infection. The outcomes of this research will provide further insight into host–virus interactions on a molecular level, which can lead to the development of effective drug and vaccine candidates aimed at interfering with the pathology caused by the virus.

## 2. Results

### 2.1. hVECs Support ZIKV Replication and Viral Production in Vitro

**Figure 1:**
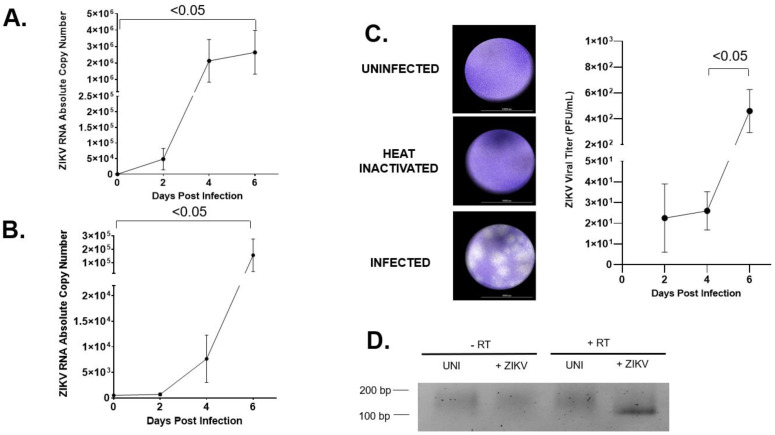
hVECs support ZIKV replication and viral production. (**A**) VK2 cell was infected with ZIKV-PR at a low multiplicity infection of 0.01. At days 0, 2, 4, and 6, positive-sense viral RNA were extracted from cell lysate and subjected to qRT-PCR analysis to monitor the dynamics of the vRNA. (**B**) Extracellular viral release from the culture supernatant of the same infected cells as in (A) was measured using qRT-PCR. (**C**) PFU viral titer from the supernatant of ZIKV-infected VK2 cells at 2, 4, and 6 days post-infection were quantified and plotted. (**D**) Negative-sense viral RNA, which is a molecular marker for de novo viral replication, was analyzed from infected cell lysates harvested at day 6 post-infection using RT-PCR. The PCR product of negative-sense vRNA was located at 102 bp. The *t* test was used to measure *p*-values.

To determine whether hVECs are permissive of ZIKV infection, a time course study was performed to measure active viral replication and production using an HPV-transformed vaginal epithelial cell line VK2E6E7 (VK2) [90]. In this study, VK2 cells were infected with the contemporary ZIKV strain PRVABC59 (ZIKV-PR), an isolate that was the causative agent for the 2015 ZIKV epidemic [7]. First, we evaluated intracellular viral RNA in the cell lysates of ZIKV-infected VK2 cells using reverse transcription (RT) PCR and real time PCR. Using real time PCR, positive-sense viral RNA transcripts were quantified at different days post-infection (dpi; days 0–6). No detectable levels of viral RNA were observed in the uninfected VK2 cells. Conversely, ZIKV-infected VK2 cells showed detectable levels of viral RNA copy numbers as early as two dpi, followed by a 100-fold increase in viral RNA at four and six dpi (Figure 1A). Next, we monitored extracellular viral release by determining the absolute viral copy numbers in the supernatant of ZIKV-infected hVECs at similar time points. The numbers of viral RNA transcripts in ZIKV-infected VK2 cells markedly increased throughout the course of infection, reaching 10^5^ RNA copies in culture at six dpi (Figure 1B). We then determined whether viral particles produced by VK2 cells remained infectious. In a standard plaque assay, no visible plaques were formed from Vero cells when inoculated with the supernatant of uninfected and ZIKV-heat-inactivated infected VK2 cells. However, the supernatant from the ZIKV-infected VK2 cells showed multiple plaque formations when inoculated with Vero cells (Figure 1C). We also quantified the viral titer from 2 to 6 dpi to monitor any changes throughout the course of infection. Our data showed detectable levels of plaque-forming units (PFU) at two dpi followed by a marked increase by day six. This finding validates that hVECs challenged by ZIKV resulted in increased production of infectious viral particles. Interestingly, results from RT-PCR analysis confirmed detectable levels of negative-sense viral RNA in VK2 cells challenged by ZIKV compared to uninfected ones (Figure 1D), indicating active de novo viral replication in these target cells.

**Figure 2:**
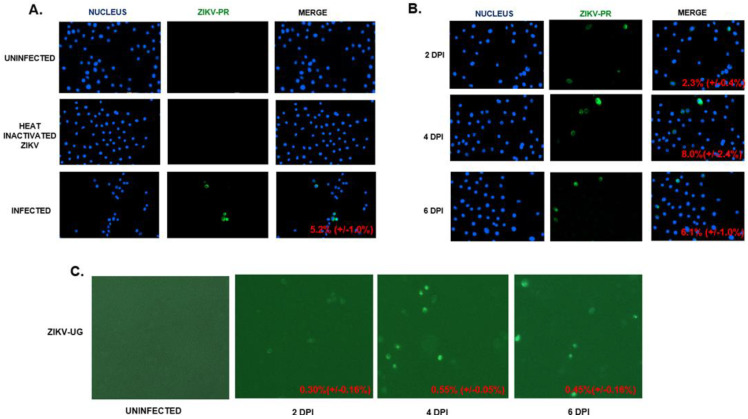
hVECs support ZIKV replication and viral production. (**A**) VK2 cells were infected with ZIKV-PR (MOI: 0.5). Uninfected and heat-inactivated ZIKV cells were used as controls. Viral replication was monitored through the presence of the viral envelope (E) protein using an indirect immunofluorescence assay (IFA) with the 4G2 primary antibody and fluorescein isothiocyanate (FITC)-conjugated secondary antibody. DAPI was used to stain the nucleus. (**B**) VK2 was infected with ZIKV-PR (MOI: 0.5). The time course of viral replication was analyzed using IFA, as mentioned above. (**C**) VK2 cells were infected with ZIKV-UG (MOI: 0.5). Venus fluorescent protein expression cells were visualized with a BioTek LionHeart FX Automated Microscope. The percentages of cells positive for ZIKV-PR were calculated by dividing the average number of ZIKV-infected cells (GFP signal) by the average number of cells. The calculated percentages are located at the lower right corners of the merged images. The total numbers of VK2E6E7 cells expressing the Venus fluorescent protein through ZIKV-UG were quantified using the BioTek LionHeart FX Automated Microscope.

The permissiveness of ZIKV in hVECs was further analyzed through an indirect immunofluorescence assay (IFA) using the anti-ZIKV envelope (E) protein monoclonal antibody. In this study, we infected VK2 cells with the contemporary Puerto Rico ZIKV strain PRVABC59 (ZIKV-PR) at a multiplicity of infection of 0.5. Staining for ZIKV E protein was not detected in uninfected and ZIKV heat-inactivated VK2 cells (Figure 2A). In contrast, the viral E protein was detectable at three dpi, confirming hVECs as a potential target cell for active ZIKV production. To assess the dynamic of viral production, we performed the same infection on VK2 cells at different time points post-infection (days 2–6). Our results indicated a marked increase in ZIKV E protein expressing VK2 cells from two (2.3 percent) to four dpi (8.0 percent), followed by a marked decrease (6.1 percent) at six dpi (Figure 2B). VK2 cells were later infected with ZIKV-UG, which expresses Venus fluorescent protein, and infected cells were monitored throughout the course of infection by Venus fluorescence (Figure 2C). Similarly to ZIKV-PR, our results indicated that active viral production in ZIKV-UG-infected VK2 cells was detectable from two to six dpi in culture. Lastly, we determined whether ZIKV infection in hVECs can be regulated under hormonal conditions. Numerous studies support the finding that sex hormones play a critical role in regulating host susceptibility and immune response against sexually transmitted viral infections [60,91,92,93,94]. It is important to consider that this immunological event has been shown to occur under various conditions, including: (1) varied detection of infection mediated by different phases of the menstrual cycle, (2) increased susceptibility to infection during pregnancy, and (3) clinical evidence showing hormonal contraception predisposes individuals to infection. To test this, different concentrations of medroxyprogesterone acetate (MPA), a progesterone derivative and hormonal contraceptive, were used to stimulate VK2 cells prior to ZIKV challenge. Since ZIKV-UG established productive infection in VK2 cells (Figure 2C), we performed this experiment using this strain. VK2 cells expressing the Venus fluorescent protein, a biomarker for ZIKV-UG infection, were monitored at three and seven days post-infection using fluorescent microscopy. Our results did not show any significant change in the average cell count among infected cells regardless of increasing concentrations of MPA (Supplemental Figure S1). This finding is interesting considering MPA has been shown to enhance HIV transmissibility in human vaginal epithelial cells in vitro [93]. Collectively, our findings underscore the need to address the regulatory role of MPA and host susceptibility among other globally relevant viruses that can establish infection via sexual transmission routes.

### 2.2. hVECs Do Not Exhibit Significant Cytopathic Effects following ZIKV Infection

**Figure 3:**
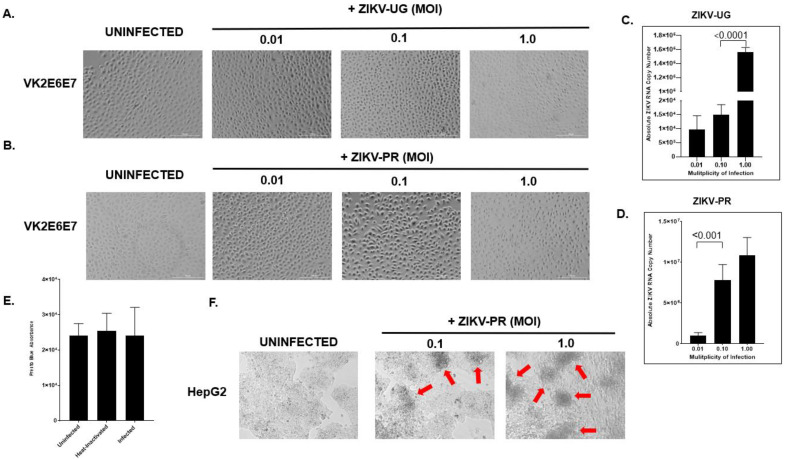
hVECs do not exhibit significant cytopathic effects following ZIKV exposure. VK2 cells were exposed to ZIKV-UG (**A**) or ZIKV-PR (**B**) at different multiplicities of infection (0.01, 0.1, and 1.0) and later examined at day six post-infection. To confirm active ZIKV infection, the supernatant from the same well shown in (**A**,**B**) was monitored using qRT-PCR. The viral RNA copy numbers were respectively quantified (**C**,**D**). Virus-induced cytotoxicity was measured biochemically using the PrestoBlue assay. The PrestoBlueTM reagent was added to the wells of uninfected, heat-inactivated, and ZIKV-PR-infected VK2E6E7 cells, which were incubated at 37 °C for at least 10 min. The absorbance of each well was quantified using a microplate reader. The relative absorbance was calculated by subtracting the absorbance of the tested wells from the absorbance reads from the culture media alone (**E**). Data were expressed as the means ± SD of triplicate experiments. To ensure that a viral cytopathic effect occurred, ZIKV-infected VK2 cells were examined alongside ZIKV-infected HEPG2 (human liver epithelial cell line) cells using bright field microscopy. Red arrows indicate CPE formation (**F**). The t-test was used to measure *p*-values.

Previous studies have shown that various human cell types supporting ZIKV replication in vitro exhibit cytopathology following viral challenge [38,95,96,97,98,99,100,101,102,103,104]. We aimed to assess whether ZIKV infection induces a cytopathic effect (CPE) in the context of hVECs. VK2 cells were exposed to ZIKV (UG and PR strains) at different MOIs and the cell morphology was later examined at six dpi using light microscopy. In the uninfected cells, we observed typical cell morphologies for the VK2 cells in a confluent monolayer culture at day six. Surprisingly, VK2 cells exposed to ZIKV did not show characteristics of cytopathic effects in either strain. These infected cells exhibited normal morphologies and increasing cell growth in culture despite being challenged with a higher viral load (Figure 3A,B). Prolonged viral culture also proved that ZIKV does not form CPEs in VK2 cells. ZIKV replication was verified using real-time PCR, which showed detectable levels of viral RNA in the supernatant of infected cells (Figure 3C,D). Additionally, the PrestoBlue^TM^ cell viability assay was used to verify that there was no detectable cell death in ZIKV-infected VK2 cells (Figure 3E). In contrast, ZIKV formed typical CPEs in Hep G2 cells under the same infection condition (Figure 3F). This key observation prompted us to further characterize this phenotype by interrogating the innate antiviral immune response to ZIKV infection in hVECs.

### 2.3. Interferon Inhibitor Ruxolitinib Enhances ZIKV Replication and Induces Cytopathic Effect in ZIKV-Infected hVECs

**Figure 4:**
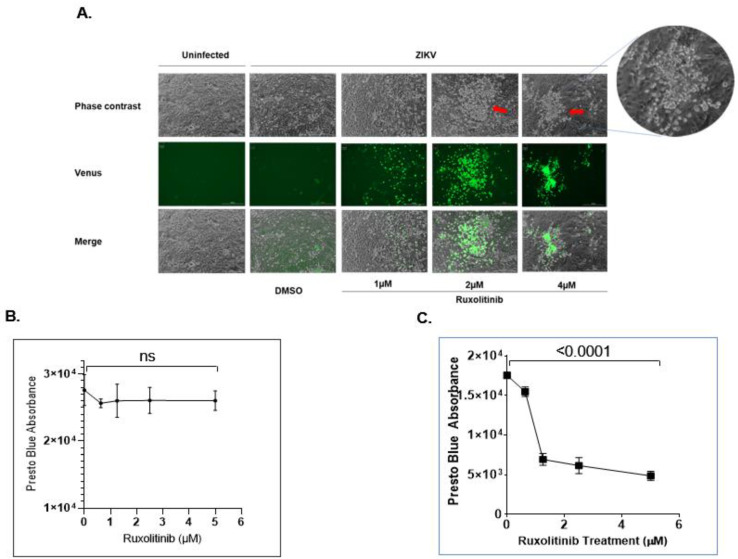
Interferon pathway inhibitor ruxolitinib enhances ZIKV replication and induces cytopathic effects in ZIKV-infected hVECs. (**A**) Different concentrations of ruxolitinib were used to treat ZIKV-UG-infected VK2 cells. DMSO treatment was used as a control. At day 7 post-infection, images were recorded using a BioTek LionHeart FX Automated Microscope. The red arrows indicate CPE formation. Cell viability for uninfected (**B**) and infected (**C**) VK2E6E7 cells treated with ruxolitinib was quantified by subjecting the cultured cells to a PrestoBlue assay. The absorbance of each well was quantified using a microplate reader. The relative absorbance was calculated by subtracting the absorbance of the tested wells from the absorbance reads from the culture media alone. The *t*-test was used to measure *p*- values. ns, not significant.

Type I IFNs play a critical role in inducing antiviral immune responses during viral infection, including in ZIKV [31,59,69,71,72,73,74,87,105]. Despite its ability to attenuate viral replication, studies demonstrate that ZIKV inhibits IFN signaling via downregulation of IRF3, NK-κB, and STAT2 [71,72,74]. Furthermore, reports show that CPE formation did occur among various cell types when infected with ZIKV, with the exception of the cells that make up the genital tract [38,95,96,97,98,99,100,101,102,103,104]. These findings may imply that the innate antiviral state of the HVT may provide protection against cytopathology during ZIKV infection. Considering this implication, we determined whether impairing the antiviral state would enhance viral replication and the resulting virus-induced CPE formation. In this study, we treated ZIKV-infected VK2 cells with ruxolitinib, a potent JAK 1/2 inhibitor, to block type I IFN response. As shown in Figure 4A, the ruxolitinib treatment increased ZIKV-UG replication in a dose-dependent manner. More importantly, CPE formation in ZIKV-UG-infected VK2 cells occurred following ruxolitinib treatment. PrestoBlue staining also showed that ruxolitinib treatment induced cytotoxicity in ZIKV-infected VK2 cells (Figure 4C) but not uninfected VK2 cells **(**Figure 4B). As expected, the uninfected cells did not show any significant change in overall cell viability post-treatment, regardless of drug concentration. These data suggest that type I IFN-mediated antiviral immune responses play a major role in inhibiting CPE formation caused by ZIKV infection.

### 2.4. IFNε Signaling Attenuates ZIKV Replication in Hep G2 Cells

**Figure 5:**
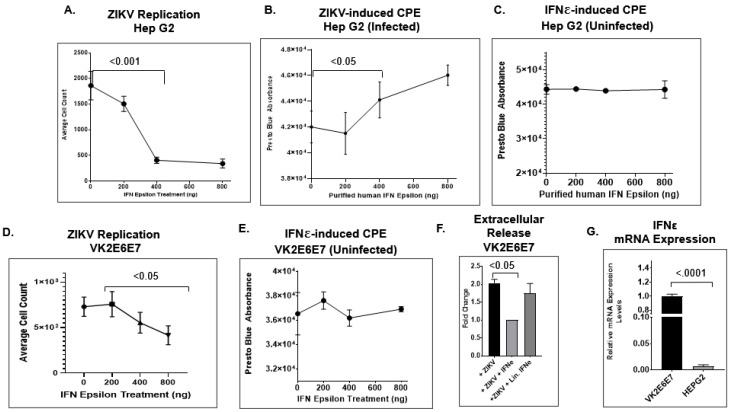
IFNε attenuates ZIKV replication. (**A**) Hep G2 cells were infected with ZIKV-UG (MOI: 0.25). Different concentrations of human IFNε were used to treat the infected cells. Five days post-infection, the Venus fluorescent protein-expressing cells were visualized using a BioTek LionHeart FX Automated Microscope. The total number of Venus fluorescent protein-expressing cells was plotted with the concentration of IFNε. Cytopathic effects from both ZIKV-infected (**B**) and uninfected (**C**) HEPG2 cells were measured using the PrestoBlue assay, in which the relative absorbance of live, viable cells was quantified using a microplate reader. (**D**) VK2E6E7 cells were infected with the ZIKV-UG strain alongside different concentrations of purified human IFNε. The average cell count was calculated by quantifying the Venus fluorescent protein-expressing cells using a BioTek LionHeart FX Automated Microscope. (**E**) The CPEs of uninfected VK2E6E7 treated with different concentrations of IFNε were measured with PrestoBlue and quantified using a microplate reader. (**F**) Extracellular viral release was measured by collecting the culture supernatant from experimental samples and quantifying the absolute viral RNA copy number using qPCR analysis. (**G**) Using qPCR analysis, the expression profiles of IFNε for the HEP G2 and VK2E6E7 cell lines were measured, and 18 s rRNA was used as a normalizing control. An unpaired *t*-test was used to measure *p*-values.

Studies have demonstrated that IFNε plays a pivotal role in mucosal immunity among various mucosal cell types. According to earlier studies, the constitutive expression of IFNε provides an antiviral state that controls the proliferation of foreign pathogens within the genital tract [75,76]. However, it is unclear whether IFNε signaling actively inhibits ZIKV replication. To answer this question, we used a different dose of IFNε protein (50 ng/mL to 800 ng/mL) to treat Hep G2 cells infected with ZIKV-UG and monitored viral replication by counting Venus-expressing cells. The HEP G2 cell line/hepatocyte was the ideal candidate for this study, considering that it exhibits the following characteristics: (1) high permissiveness and overt cytopathology during ZIKV infection (as shown in Figure 3F) [95] and (2) inefficient expression of endogenous IFNε in mammalian cells [106]. First, we evaluated the expression profile of IFNε in both HEP G2 and VK2E6E7 cell lines using qRT-PCR analysis. Our data indicated that HEP G2 significantly expressed lower levels of *IFNε* mRNA compared to the VK2E6E7 cell line (Figure 5G). Next, we wanted to determine whether endogenous IFNε levels correlated with viral replication and virus-induced CPEs in both ZIKV-infected HEP G2 and VK2E6E7 cell lines. In infected HEP G2 cells, our results showed that the average cell count of Venus-expressing cells dramatically decreased when treated with IFNε at different doses (Figure 5A). The estimated ED50 was between 200–400 ng/mL. According to the product description, the activity of the IFNε occurs at ED50 values of 100–500 ng/mL against the encephalomyocarditis (EMC) virus in Hela cells. Next, we tested whether the antiviral activity of IFNε reduced viral cytopathology by performing the PrestoBlue cell viability assay with both infected and uninfected HEP G2 cells treated with IFNε. In uninfected HEP G2 cells, our results did not indicate any significant change in cell viability in culture (Figure 5C). Conversely, ZIKV-infected HEP G2 cells did show a marked increase in cell viability under IFN treatment conditions in a dose-dependent manner (Figure 5B). This result shows that the biological role of IFNε during ZIKV infection is one of promoting attenuation of viral replication and viral-induced cytopathology. Furthermore, our findings provide new insights into one of the contributing factors responsible for the viral replication dynamics observed in our study. Lastly, we aimed to test the antiviral activity of IFNε signaling in hVECs by infecting VK2E6E7 cells with the ZIKV-UG strain at different concentrations of purified IFNεs. At a dose-dependent level, the total Venus fluorescent protein-positive cells showed a marked decrease at increasing IFNε concentrations (Figure 5D). Interestingly, the decrease in ZIKV-positive cells in VK2E6E7 cells was not as dramatic as in ZIKV-infected HEP G2 cells treated with IFNε protein. This inverse relationship further validated the antiviral effects of IFNε during ZIKV infection. We also performed the PrestoBlue assay to analyze the potential effects of IFNε signaling in VK2 cells during infection. As expected, we did not see any significant changes in cell viability from treating the cells with the purified cytokine (Figure 5E). Apart from ZIKV replication, we also determined whether the antiviral activity of IFNε affects extracellular release in ZIKV-infected VK2E6E7 cells. Culture supernatant from ZIKV-infected VK2E6E7 cells treated with either IFNε or linearized IFNε (treatment control) were collected and later subjected to qRT-PCR analysis for viral RNA quantification. At five days post-infection, our results indicated that IFNε treatment significantly decreased extracellular viral release in VK2E6E7 cells (Figure 5F).

### 2.5. IFNε Treatment Dampens ZIKV Replication in hVECs via Induction of Type I Interferon-Stimulated Genes

**Figure 6:**
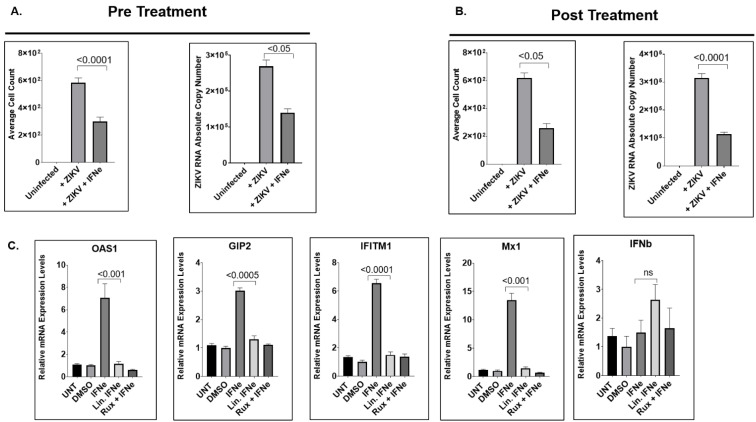
IFNε treatment restricts ZIKV replication via induction of Type I Interferon stimulated genes. (**A**) Effect of pretreatment with IFN-ε on ZIKV infection in hVECs. VK2E6E7 cells were stimulated with recombinant IFN-ε for one hour prior to ZIKV infection with the ZIKV-UG strain. Viral replication and extracellular release were measured at five days post-infection. (**B**) Antiviral effects of IFN-ε treatment after ZIKV infection. VK2E6E7 cells were infected with ZIKV-UG and later treated with IFN-ε after infection. (**C**) IFN-ε induces type I IFN-stimulated genes (ISGs). VK2E6E7 cells were treated with IFN-ε for six hours, and gene expression of ISGs was measured by real-time qPCR analysis. Relative mRNA expression levels (as fold change relative to DMSO control) for each gene of interest were calculated using the ΔΔCT method, as described in the Materials and Methods Section. The normalizing control used for the qPCR was the housekeeping *GUSB* gene. An unpaired t-test was used to analyze the *p*-values for the viral replication and induction of ISGs.

We aimed to further characterize the antiviral effects of IFNε on ZIKV infection of hVECs under pre-treatment and post-treatment conditions. Under pre-treatment conditions, the average numbers of ZIKV-positive cells and extracellular viral RNA in collected supernatants showed significant reductions in VK2E6E7 cells treated with IFNε compared to no treatment (Figure 6A). Similarly, VK2E6E7 cells treated with IFNε showed reductions in overall ZIKV infection under post-treatment conditions (Figure 6B). Next, we aimed to demonstrate the ability of IFNε to activate type I IFN signaling in human vaginal epithelial cells. Interestingly, VK2E6E7 cells treated with IFNε showed significant increases of the type I IFN-stimulated genes *oas1*, *mx1*, *g1p2*, and *ifit1* within 6 h post-treatment (Figure 6C) when compared to linearized IFNε. Interestingly, the expression levels of *ifnb* did not show any significant change when treated with IFNε. Collectively, our data indicate that IFNε uniquely limits active ZIKV replication and production in hVECs via type I IFN signaling.

### 2.6. Primary Human Cervical Cells Are Susceptible to ZIKV Infection

**Figure 7:**
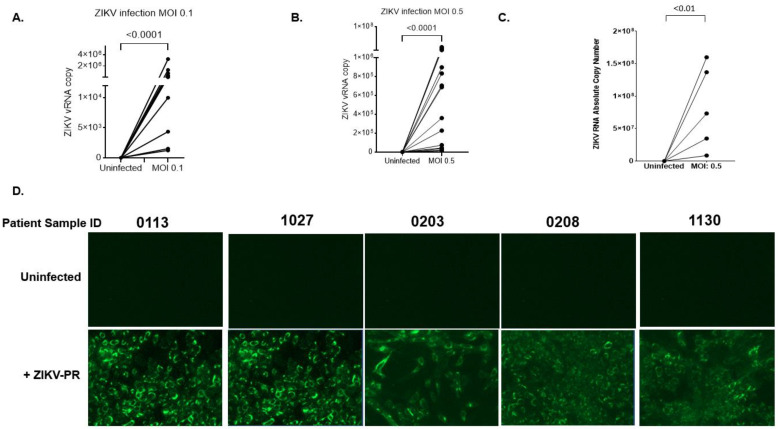
Primary human cervical cells are susceptible to ZIKV infection. (**A**,**B**) Primary human cervical cells from 15 donors were infected with ZIKV-PR (MOI: 0.1 or 0.5). After infection, the cells were extensively washed and culture for four days. Four days post-infection, the culture supernatant was subjected to qRT-PCR to measure viral RNA copy numbers. (**C**) To determine infectivity, the cultured supernatant collected from the primary cells infected with ZIKV-PR (n = 5) was used as inoculum to infect Vero cells. The absolute RNA copy number was determined by subjecting the supernatant collected from Vero cells to qPCR analysis. (**D**) In addition to qPCR analysis, active viral production was examined by performing IFA on Vero cells infected by the inoculum. The Wilcoxon matched-pairs signed-rank test was used to analyze the significance of viral replication.

To further confirm that ZIKV replicates in the vaginal cell type are physiologically relevant, it was essential to test whether ZIKV replicates in primary human cervical epithelial cells (hCECs). To do this, we analyzed the viral release from primary human cervical epithelial cells infected with ZIKV-PR. Using the contemporary ZIKV-PR strain, primary hCECs were infected at multiplicities of infection of 0.1 and 0.5 over a course of four days. Due to limited tissue availability, we were unable to perform IFA to verify active viral E protein production using the 4G2 monoclonal antibody. Nevertheless, as shown in Figure 7A,B, the extracellular viral release was measured by quantifying the viral RNA copy number from the supernatant of infected cells. Similar to the viral kinetics in the VK2 cell line, our results showed that the primary hCECs from 15 independent patient donors actively supported ZIKV infection in culture. Detectable levels of viral RNA were present in the supernatant of primary cells infected with ZIKV-PR at MOIs of 0.1 and 0.5. Each patient sample showed a varying range of extracellular viral particles, which was deduced based on absolute viral RNA copy number readings from 10^2^–10^6^ obtained from qPCR analysis. The Wilcoxon matched-pairs signed-rank test showed that viral release from infected cells was very significant (*p* < 0.0001 for both MOI 0.1 and 0.5) comparing to uninfected ones. We further examined whether these viral particles produced from the primary cervical epithelial cells remained infectious. Supernatant collected by five donors in this study was used as inoculum for infecting Vero cells in culture. Viral infectivity was later analyzed by performing qPCR analysis and IFA at four days post-infection. Our results indicated detectable ZIKV RNA levels from Vero cells inoculated with the supernatant from the infected donor samples (Figure 7C). Similarly, the inocula collected from the infected donor samples resulted in detectable levels of ZIKV E protein in Vero cells. No detectable levels of ZIKV RNA, or ZIKV E protein were present in Vero cells inoculated with the supernatant from uninfected donor samples (Figure 6D). These results collectively support our hypothesis that the outermost epithelial layer located in the vaginal tract serves a target cell for local ZIKV replication.

## 3. Discussion

ZIKV has been a major public health concern since the 2015 outbreak in the United States [5,7,13]. Typically, a large percentage of clinical patients infected with ZIKV do not exhibit symptoms characteristic of clinical disease [14,15,16]. The remaining percentage exhibit mild fever accompanied by skin rash, conjunctivitis, muscle and joint pain, and malaise. The alarming issue with Zika clinical disease is the strong link between fetal abnormalities and neurological complications, including microcephaly. The Centers for Disease Control and Prevention (CDC) reported over 200 US cases of live and dead infants with severe fetal defects, including placental deficiency, stillbirth, and spontaneous abortion [107]. In infected fetuses, some cases involved brain atrophy, coarse calcification, and other microcephaly-associated symptoms. During the ZIKV outbreak in French Polynesia, Brazil, and Puerto Rico, there was an increase in the incidence of Gullian-Barré syndrome (GBS), an autoimmune neuropathy in which damaged neurons result in muscle weakness and limb paralysis [14,15].

Despite this growing evidence associating ZIKV with sexual transmission, the underlying molecular mechanism of ZIKV pathogenesis, particularly in the human vaginal epithelium, remains elusive. The HVT, which consists of the vagina and cervix, is covered by an epithelial layer that serves as a physical and mechanical barrier against viruses [63,108]. Some viruses, however, exploit this epithelial layer as a portal of entry into the human host. In the context of vaginal intercourse with an infected partner, the potential target cells for ZIKV infection are likely to be localized to the outermost epithelial layer of the vaginal tract. Although recent studies demonstrate that ZIKV exposure in the vaginal tract results in productive infection and vaginal transmission [55,56], the molecular interactions between host innate immunity and local viral replication, specifically in the human vaginal epithelium, remain to be explored. Our study uniquely attempts to address this gap in knowledge by performing a time-course kinetics study to measure active viral replication and production using the transformed human vaginal epithelial cell line VK2E6E7. Interestingly, our results collectively demonstrate that hVECs are a viable cell target for ZIKV to initiate de novo viral RNA replication and ZIKV E protein production. Most importantly, our findings show that ZIKV replication dynamics in hVECs are not short-lived, but rather increase for as long as six days post-infection. Lastly, the infectivity of the virus isolated from VK2 cells was evaluated using a plaque assay. The results showed the presence of visible plaques among the supernatant collected from ZIKV-infected VK2 cells compared to uninfected cells. Collectively, these results further confirm that hVECs support productive infection and viral spread, which supports our hypothesis that this cell type is a direct target cell for viral spread and dissemination within the vaginal tract.

It is important to consider that the external milieu on the surface of HVT is a mucosal layer that consist of antimicrobial peptides, commensal microbes, sex-related hormones, immunoglobulins, and residential immune cells [60]. The complexity of innate mucosal immunity within the HVT and its functional role against viral pathogens arguably pose some limitations for this study. Nevertheless, there is some progress in further closing this gap in knowledge. First, our study showed that stimulating hVECs with a hormonal contraceptive MPA did not significantly regulate the susceptibility of hVECs to ZIKV infection, even at increasing concentrations (Supplemental Figure S1). These data seem to contradict previous studies that demonstrate that MPA does enhance transmissibility, particularly in HIV [93] and HSV-1 [109]. Other than sex hormones influencing viral replication, one study from Pyles et al. used an ex vivo, multilayer vaginal epithelial cell culture system to determine that the colonization of commensal bacteria containing *Staphylococcus* spp. reduced ZIKV replication [53]. Despite these findings, mechanisms involving the innate antiviral state of HVT and ZIKV pathogenesis still remain elusive. It would be beneficial to investigate these molecular mechanisms by incorporating ex vivo and/or in vitro model systems that recapitulate the external milieu of the vaginal mucosae.

Viral infections of permissive cells are usually associated with changes in cell morphology and cell physiology and biochemical events [110]. During the course of infection, these changes in the infected cell can either result in cell death or normal cell growth over long periods of time. To determine if ZIKV induces cytopathic effects (CPEs) in the human vaginal epithelium, we exposed VK2 cells to ZIKV and later monitored characteristics of virus-induced cytotoxicity using light microscopy. Interestingly, no significant changes were observed among infected cells compared to uninfected cells throughout the course of infection. However, this observation presented limitations in terms of drawing conclusive evidence that these events occurred. We validated this by performing a PrestoBlue assay to test cell viability. Our results showed no significant differences in the relative absorbance between the ZIKV-infected and non-infected cell samples. This finding is significant considering that various human cell types do show CPEs and/or undergo structural changes during ZIKV infection [38,95,96,97,98,99,100,101,102,103,104]. It was thus considered imperative to further investigate whether the innate antiviral state within hVECs uniquely contributes towards attenuating viral replication.

The outcome of viral infection is determined by a competition between viral virulence factors and host innate antiviral immunity [60,61,69,70,73,75,105,111]. During viral invasion, the exposed viral RNA initiates activation of sensory molecules that control local replication and limit systemic infection within the host. Recognition of these non-self-nucleic acids triggers a signaling cascade that results in the induction of antiviral gene products. Current reports have made progress in determining that signaling pathways of types I and III play a vital role in the innate immune response against ZIKV [71,72,112]. Numerous studies have shown that ZIKV interacts with sensory molecules, such as toll-like receptor 3 (TLR3) and retinoic acid inducible gene 1 (RIG-1) [113], as well as the transcription factors interferon regulatory factor 3 (IRF7) and NFκ-B, following ZIKV infection [71]. These molecules serve as important factors in limiting RNA viral replication and viral spread, including for ZIKV. Despite evidence linking type I IFNs to viral attenuation, many of these studies only characterize the conventional type I IFNs α and β, which are exclusively inducible via the activation of pathogen pattern recognition receptors (PRRs) [70,73,105]. Furthermore, several in vitro studies have demonstrated that ZIKV reduces the activity of host innate antiviral immunity by antagonizing type I IFN production and signaling [31,59,71,72,74]. What remains to be explored are the remaining type I IFNs that play unique antiviral roles in the event of viral infection, especially in the HVT. Tissues that are specific to mucosal sites, such as the HVT, constitutively express IFNε, a hormonally regulated cytokine that is not induced by known PRRs [75,77,81,86]. Similar to conventional IFNs α and β, IFNε has been shown to directly mediate protection against the dissemination of foreign pathogens [75,76,89]. It is important to note that the biological activity of IFNε is relatively low for a type I IFN and it exhibits low binding affinity for IFNAR receptors [77]. It is hypothesized that the low activity of IFNε may be advantageous to specific tissues by limiting the potential toxicity typically associated with conventional IFNs. Moreover, the low activity enables constitutive expression of IFNε in mucosal tissues, limiting the internalization of the IFNAR receptor that would render these cells refractory over time.

Unfortunately, there is a huge gap in knowledge as to how IFNε-mediated signaling responds to infections from other globally relevant viruses, especially flaviviruses. Our study took the initiative in filling this gap by examining the role of IFNε during active ZIKV replication. Our results show that IFNε inhibits ZIKV replication at ED50 < 300 ng/mL. Additionally, we found that IFNε induces several type I IFN-stimulated genes in hVECS, including *oas1*, *mx1, g1p2*, and *ifit1*. The collective results underscore how the innate antiviral state mediated by IFNε signaling uniquely attenuates active ZIKV replication in the human vaginal epithelium. Despite its antiviral activity in hVECs, it is important to note that IFNε activity only reduce, not eliminate, ZIKV replication from these target cells. Taking this into account, it can be speculated that the innate antiviral state mediated by IFNε may inadvertently promote viral spread and dissemination in the human vaginal epithelium [114]. In other words, infecting the human vaginal epithelia may serve as an effective transmission strategy by allowing ZIKV to actively replicate in an immune-competent site while not inducing a robust host immune response sufficient for viral clearance. Given that, our research findings provide novel insight into a mechanism by which ZIKV can successfully replicate in target cells, such as the human vaginal epithelium, despite the presence of a sustained innate antiviral environment via IFNε signaling.

## 4. Materials and Methods

Cells, virus, plasmids, reagents and antibodies. The VK2/E6E7 human vaginal epithelium cell line (ATCC CTL-2616) was cultured at 37 °C and 5% CO_2_ in keratinocyte serum-free medium (SFM) (ThermoFisher) supplemented with recombinant epidermal growth factor (0.1 ng/mL), bovine pituitary extract (50 μg/mL), and calcium chloride (0.4 mM). The 293T human kidney epithelial cell line (ATCC CRL-3216) and the Vero green African monkey kidney cell line (ATCC CCL-81) were cultured in DMEM medium (ThermoFisher) plus 10% FBS. The Hep G2 human liver epithelial cell line (ATCC HB-8065) was cultured in low-glucose DMEM (ThermoFisher Cat# 11885-084) with 10% FBS. The low-passage-number ZIKV strain PRVABC59 (termed ZIKV-PR hereafter) was a gift from Dr. Brandy Russell at the CDC. This particular strain was isolated from a viremic patient in Puerto Rico in 2015. Vero cells were used for virus propagation and are routinely used in ZIKV research. The Uganda ZIKV prototype strain MR766 (ZIKV-UG) was generated using a plasmid carrying a cytomegalovirus (CMV) promoter-expressed 1947 Uganda MR766 prototype ZIKV cDNA clone, courtesy of the Evans lab at the Mount Sinai School of Medicine. The multicycle kinetics curve and plaque assay experiments verified that the plasmid-based MR766 virus exhibited similar growth patterns to its natural parental isolate [115]. In accordance with the methods described by Schwarz et al., the plasmid-based ZIKV-UG strain was produced in 293T cells and the produced virions were amplified using Vero cells. ZIKV inactivation was achieved by heating virus stock at 100 °C for 5 min, which was later stored at −80 °C until further experimentation. Primary human cervical epithelial cells were isolated from de-identified female genital tract tissues provided by the Cooperative Human Tissue Network (CHTN)—Western Division at Vanderbilt University Medical Center and by the Meharry Medical College Translational Pathology/Tissue Acquisition Shared Resource, as previously described [93]. The sample collection protocol was approved by the Meharry Medical College IRB. Ruxolitinib (cat #: S1378) was purchased from Selleck Chemicals. Recombinant human IFN epsilon protein (cat #: 9667-ME-025) was purchased from R&D Systems. Monoclonal antibody 4G2 against ZIKV envelope protein was from the tissue culture of a D1-4G2-4-15 mouse hybridoma cell from ATCC (HB-112). The RNA copy number standard for qRT-PCR, quantitative synthetic RNA from the Zika virus (cat #: VR-3252SD), was purchased from the ATCC.

ZIKV infection of cells. Cells were seeded in culture plates with a growth area of 2 × 10^4^ cells/cm2 (12-well or 48-well plates). The cells were then exposed to ZIKV at the desired multiplicity of infection (MOI) and incubated for 1–2 h with gentle agitation for efficient viral adsorption. Next, the inoculum was removed and washed with phosphate-buffered saline (PBS) three times. Fresh culture medium was added to each well and incubated at 37 °C and 5% CO_2_ for the duration of the experiment.

Interferon epsilon protein treatment. A series of different concentrations of recombinant human IFN epsilon protein, ranging from 50 ng/mL to 800 ng/mL, were added to the cell culture during the infection of Hep G2 cells with ZIKV-UG after 1–2 h of viral adsorption. The same volume of culture medium was added as an untreated control. After washing, fresh culture medium with the same concentrations of IFN epsilon protein was added into the tissue culture. The cells expressing Venus fluorescence protein were counted using a BioTek Lionheart FX Automated Microscope. Linearized human IFN epsilon was prepared by heating protein stock at 100 °C for 5 min. The protein stock was then stored at −80 °C for future experiments.

Interferon pathway inhibitor treatment. A series of different concentrations of ruxolitinib [116], ranging from 1 µM to 4 µM, were used to treated VK2/E6E7 cells infected with ZIKV-UG during the 1–2 h viral adsorption step. The DMSO-treated one sample used as an untreated control. After washing, fresh culture medium with the same concentrations of ruxolitinib was added into the tissue culture. A BioTek Lionheart FX Automated Microscope was used to observe viral plaque formation and Venus-expressing cells.

Medroxyprogesterone acetate (MPA) treatment. Different MPA (Sigma Aldrich) concentrations, ranging from 1 µM to 8 µM, were used for the treated VK2/E6E7 cells infected with ZIKV-UG during the 1–2 h viral adsorption step. The DMSO treated sample was used as an untreated control. After washing, fresh culture medium was added into the cell culture for the indicated time periods post-infection. A BioTek Lionheart FX Automated Microscope was used to observe the Venus-expressing cells. This method was adapted from the protocol described in the MPA by Jia et al. [93].

Negative-sense RNA RT-PCR. Strand-specific RT-PCR was performed to verify the presence of negative-strand ZIKV RNA, a marker of de novo viral replication in infected cells. Intracellular viral RNA was extracted from the cell lysates harvested using the TRIzol^®^ (Life Technologies, Carlsbad, CA, USA) phase separation method. Briefly, 250 µL of TRIzol^®^ reagent was added to lyse the cells, and the RNA was isolated via phase separation by adding 50 µL chloroform. After phase separation, the lysate was gently vortexed and centrifuged at 4 °C to extract the RNA from the sample. The RNA was later further purified with an Aurum Total RNA Mini Kit (Bio-Rad Laboratories, Hercules, CA, USA). After washes, the RNA was eluted, aliquoted, and stored at −80 °C prior to RT-PCR. cDNA synthesis was conducted using a High-Capacity cDNA Reverse Transcription Kit (ThemoFisher Scientific, Waltham, MA, USA) with a primer (5′-CATTGGTAACCGCATTGAAA-3′) specifically annealed to the negative-sense viral RNA. cDNA generated from the samples were later amplified using the Hot Start *Taq* DNA polymerase kit (New England Biolabs^®^, USA) with the forward primer 5′-AARTACACATACCARAACAAAGTGGT-3′ and reverse primer 5′-TCCRCTCCCYCTYTGGTCTTG-3′. Four microliters of cDNA alongside 10× standard *Taq* reaction buffer, 10 mM dNTPs, 10 µM forward and reverse primer, Hot Start *Taq* DNA polymerase, and nuclease-free water were used to create the master mix necessary for DNA amplification. The PCR cycling conditions were 95 °C for 30 s, followed by 30–35 cycles of 95 °C for 30 s, 56 °C for 1 min, 68 °C for 30 s, and a final extension of 68 °C for 2.5 min. The 102 bp PCR products were observed using gel electrophoresis.

ZIKV qRT-PCR. Total RNA extraction from culture supernatants and cDNA synthesis was carried out as described above using a random hexamer primer. After cDNA synthesis, each sample was analyzed in a Bio-Rad CFX96 Real-Time PCR system using SYBR Green technology (Bio-Rad Laboratories, USA). Each 8.5 μL reaction mixture contained 100 nM of the forward primer 5′-AARTACACATACCARAACAAAGTGGT-3′ and reverse primer 5′-TCCRCTCCCYCTYTGGTCTTG-3′ and a 1x final concentration of SYBR Green florescent dye. Amplification conditions were 40 cycles of 95 °C for 3 min followed by 95 °C for 15 s and 60 °C for 1 min. Quantitative synthetic RNA from Zika virus (ATCC VR-3252SD) was used as a standard to generate a standard curve. The viral RNA copy number was calculated based on the standard curve.

Immunofluorescent assay (IFA). Two to six days following infection, ZIKV-infected and uninfected cells on coverslips were fixed with 3.8% paraformaldehyde in 1× PBS for 15 min. After fixation, cells were permeabilized (0.1% Triton 100 and 0.2% bovine serum albumin (BSA) in 1× PBS) for an additional 15 min. Next, cells were washed in PBS and blocked by incubation in 5% BSA for 30 min. Afterwards, cells were incubated for 60 min at room temperature using the desired primary antibody and secondary antibody with the desired conjugated fluorophore. Lastly, DAPI dye was used to stain the nucleus. A BioTek Lionheart FX Automated Microscope was used to observe each coverslip.

ZIKV plaque assay. Four tenfold serial dilutions of ZIKV samples were exposed to monolayers of Vero cells at 37 °C for two hours. Cells were washed in PBS and cultured in DMEM supplemented with 10% FBS and 1.0% low-melt agarose. The cells were maintained at 37 °C for 6 days until they were fixated with 3.7% formaldehyde. After fixation, cells were stained with crystal violet solution (0.1% crystal violet in 20% ethanol solution) for 4 h, and then visible plaques were counted to quantity viral titers. This experiment was repeated three times.

PrestoBlue assay. Cell viability after ZIKV infection was determined using a PrestoBlue cell viability kit (Invitrogen, Waltham, MA, USA). In brief, the human vaginal epithelial cell line VK2 was seeded into a 96-well plate and incubated overnight. Cells were later inoculated with the ZIKV-PR strain (PRVABC59), heat-inactivated virus inoculum, or neither (uninfected) and treated with different concentrations of both drugs either alone or in combination and incubated for 48 h. Six days post-infection, fresh culture medium was added into the respective wells, and the PrestoBlue reagent was added and the medium incubated for at least 10 min. Measurement of fluorescence (540 nm excitation/590 nm emissions) was quantified using a ClarioStar^®^ microplate reader (BMG Labtech, Cary, NC, USA). Relative absorbance was calculated by subtracting the raw reads from the tested wells from the absorbance reads taken from the media alone. Data were expressed as the means ± SD of triplicate experiments.

Statistical analysis. Unless specified, experiments were repeated at least three times with appropriate controls. *p*-values were calculated with the Wilcoxon matched-pairs signed-rank test and t-tests using GraphPad Prism.

## 5. Conclusions

We identified human vaginal epithelial cells, which serve as the first defensive line against foreign pathogens, as the direct target cells for ZIKV vaginal infection. In this study, we determined that hVECs exposed to ZIKV resulted in active de novo *RNA* replication and viral E production, as well as extracellular release of infectious viral particles. Our research findings further indicated that the replication dynamics of ZIKV in hVECs did not result in significant changes to the overall cell morphology and cell growth when grown in culture. Most importantly, we highlighted the dual aspects by which IFNε and active IFN signaling assist in the attenuation of ZIKV replication and conferment of viral dissemination. Based on our collective finding, we propose that the mechanism of ZIKV sexual transmission is established by ZIKV directly infecting the outermost epithelial layer of the vaginal tract. Furthermore, the immune-privilege site of hVECs allows ZIKV to disseminate through the epithelium successfully without being cleared by robust immune responses. Identifying this mechanism of transmission will be instrumental for the development of drug and vaccine candidates aimed at disrupting the pathology caused by the virus.

## Data Availability

The data presented in this study are available in the paper and supplementary material.

## Supplementary Materials

The following supporting information can be downloaded at https://www.mdpi.com/article/10.3390/pathogens11080853/s1.
