# Supplementary material for: Interferon Epsilon Signaling Confers Attenuated Zika Replication in Human Vaginal Epithelial Cells
Source: Pathogens. 2022 Jul 29;11(8):853. doi: 10.3390/pathogens11080853 (PMC9415962; doi:10.3390/pathogens11080853)
Supplement: Supplementary file 1 [file pathogens-11-00853-s001.zip › pathogens-1651150-supplementary.pdf]

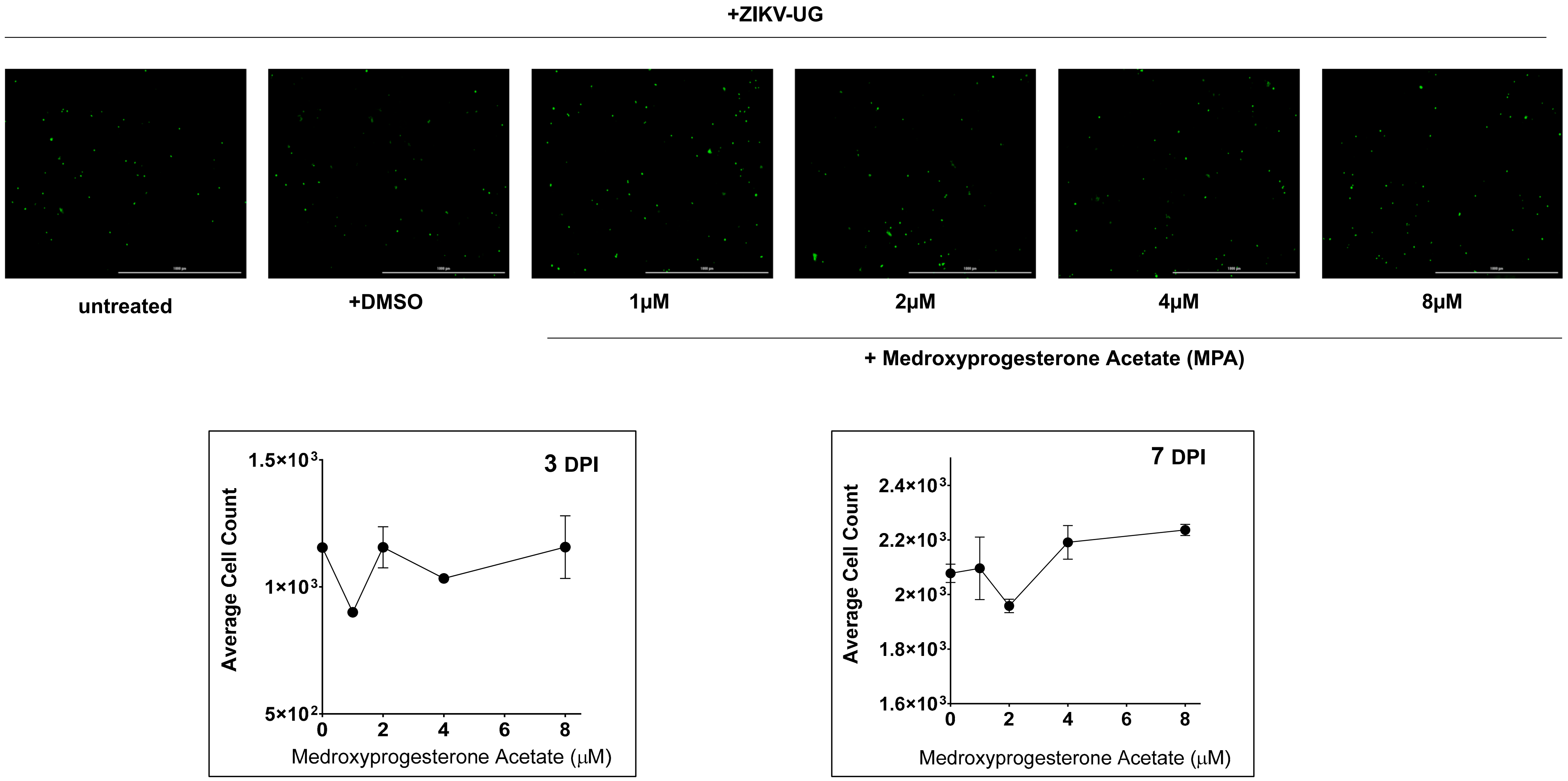

Supplemental Figure S1. Progesterone Derivative Medroxyprogesterone Acetate (MPA) Did Not Influence Viral Infection in ZIKV-infected hVECs. (A) Different concentrations of Medroxyprogesterone was used to treat ZIKV-UG-Venus infected VK2 cells. Untreated and DMSO treated were used as controls. Day 3 and 7 post-infection, imaging was taken using the BioTek LionHeart FX Automated Microscope. Average cell count was measured by quantifying Venus-expressing VK2E6E7 cells, which is a biomarker for active viral production.

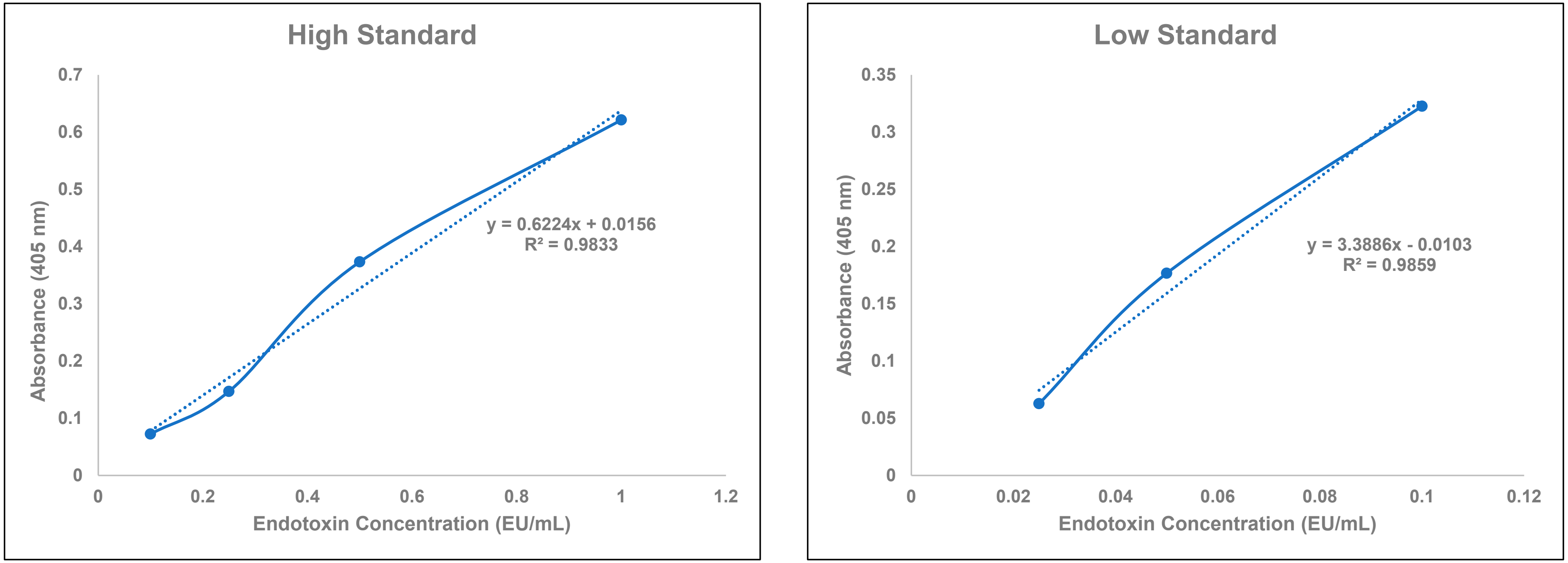

Supplemental Figure S2. Endotoxin Levels of purified IFNε stock. (**A**) To ensure that the antiviral effect isn't from LPS contamination, the endotoxin levels in the IFNε stock used in the experiments were quantified using Pierce™ Chromogenic Endotoxin Quant Kit. Endotoxin levels in the samples are accurately determined using an endotoxin standard (high standard or concentration and low standard or concentration) of known concentration that is derived from E.coli strain O111: B4. The absorbance of each wells were quantified using a microplate reader. The relative absorbance was calculated by subtracting the absorbance of the tested wells minus the absorbance reads from the endotoxin free water alone. The average absorbance (405 nm) measured from the IFNε stock was 0.018, which correlates to an endotoxin concentration of <.01 EU/mL. Each standard and experimental samples used in the experiment were done in triplicates.
